# Cytomegalovirus (CMV) Co-infection in Treatment-Refractory Immune Checkpoint Inhibitor-Associated Colitis: A Case Report

**DOI:** 10.7759/cureus.111235

**Published:** 2026-06-21

**Authors:** Michael Stanczyk, Xiaobang Hu

**Affiliations:** 1 Department of Pathology and Laboratory Medicine, Penn State College of Medicine, Penn State Health Milton S. Hershey Medical Center, Hershey, USA

**Keywords:** cmv, colitis, histology, immune checkpoint inhibitor, infection

## Abstract

Immune checkpoint inhibitors (ICIs) have been increasingly used for the treatment of various malignancies, and ICI-associated colitis is one of the most common side effects. Here we report a 55-year-old man with metastatic melanoma who was initially treated with nivolumab-relatlimab. Due to side effects, therapy was transitioned to ipilimumab, followed by combination ipilimumab and nivolumab. Ten days after the third dose of ipilimumab and nivolumab, he presented to the emergency department with nausea, vomiting, bloody diarrhea, and fever. Stool pathogen panel and *Clostridioides difficile* polymerase chain reaction (PCR) were negative. ICI-associated colitis was suspected clinically, and he was treated with steroids and then one dose of infliximab. However, his condition did not improve, and a colonoscopy was performed, which showed diffuse severe inflammation characterized by adherent blood, altered vascularity, erythema, friability, and granularity in the entire colon. Biopsy showed colonic mucosa with ulceration, cryptitis, crypt abscesses, dilated crypts with flattened epithelium, and focal crypt dropout. The histologic changes were compatible with ICI-associated colitis. Interestingly, cytomegalovirus (CMV) immunostaining showed scattered positive cells. Subsequent plasma CMV PCR was positive. The patient was treated with ganciclovir. The CMV viral load decreased after treatment, and his diarrhea improved. CMV co-infection in patients with treatment-refractory ICI-associated colitis is rare, and our case highlights the importance of clinicopathologic correlation in reaching the diagnosis.

## Introduction

Immune checkpoint inhibitors (ICIs) enhance anti-tumor immune responses by blocking inhibitory immune checkpoints on T cells and are widely used in the treatment of multiple malignancies, such as melanoma, non-small cell lung cancer, and renal cell carcinoma [1]. However, ICIs are associated with a spectrum of adverse effects affecting various organ systems, mainly including the skin, digestive system, endocrine system, and respiratory system [2]. The reported incidence of ICI-associated colitis varies, and there appears to be a higher incidence with ipilimumab-based regimens as compared with programmed cell death protein 1 (PD-1)/programmed cell death ligand 1 (PD-L1) inhibitors [3]. The management of ICI-associated colitis depends on disease severity. For moderate to severe cases, it is recommended that ICI be paused and corticosteroid therapy should be initiated [4]. However, a subset of patients can develop treatment-refractory disease [5]. In this setting, superimposed infection should be ruled out, as mucosal injury and escalating immunosuppression may predispose patients to opportunistic pathogens, including reactivation of latent viral infections such as cytomegalovirus (CMV) [5,6]. CMV infection can mimic or exacerbate ICI-associated colitis through overlapping clinical, endoscopic, and histologic features [7]. Here, we report a case of CMV co-infection in treatment-refractory ICI-associated colitis and highlight the importance of clinicopathologic correlation in reaching the diagnosis.

## Case presentation

A 55-year-old man with stage III melanoma was treated with nivolumab-relatlimab. Due to side effects, therapy was transitioned to ipilimumab, followed by combination ipilimumab and nivolumab. Ten days after the third dose of ipilimumab and nivolumab, he presented to the emergency department with inability to tolerate oral intake, persistent vomiting, profuse bloody diarrhea, low-grade fever (maximum temperature 100.7°F), and an unintentional weight loss of approximately 10 pounds. On examination, he had decreased urine output and severe diarrhea with incontinence. The bilateral lower extremity showed purple discoloration and was tender to palpation. Initial infectious evaluation was notable for a positive COVID-19 test, and the patient was treated with remdesivir. An enteric pathogen panel was negative.

**Figure 1:**
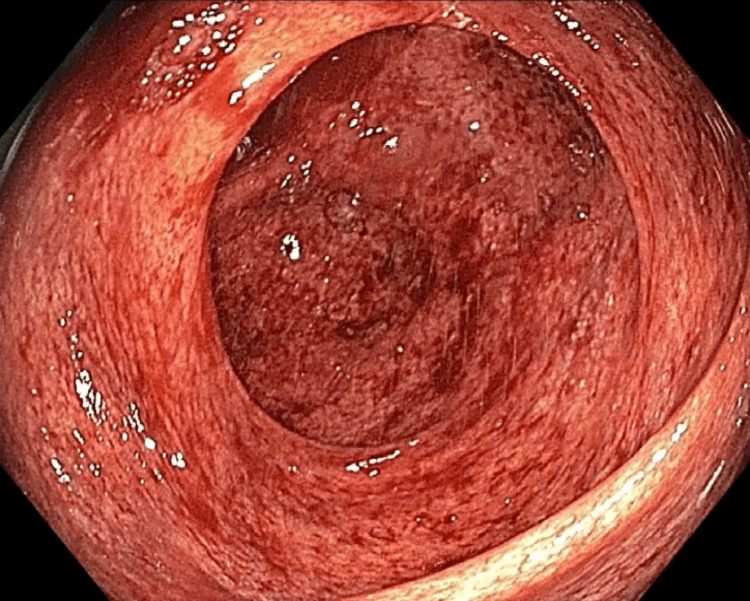
Endoscopy shows diffuse severe inflammation characterized by adherent blood, altered vascularity, erythema, friability, and granularity in the entire colon.

ICI-associated colitis was suspected clinically, and the patient was started on prednisone. Due to persistent symptoms, hepatitis B screening was performed and was negative before initiation of infliximab. Despite the therapy, the patient continued to experience significant diarrhea, and a colonoscopy was performed. Endoscopic examination shows diffuse severe inflammation characterized by adherent blood, altered vascularity, erythema, friability, and granularity throughout the colon (Figure 1). A biopsy was performed.

**Figure 2:**
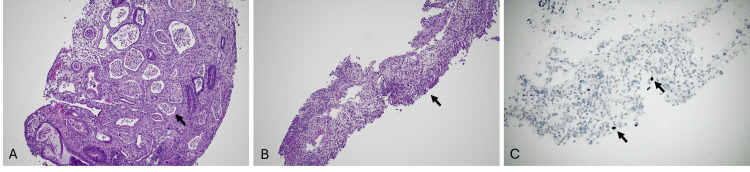
A, B. Colon biopsy shows colonic mucosa with cryptitis, crypt abscess, dilated crypts with flattened epithelium (arrow) (A, H&E stain, 100x), ulceration, and crypt dropout (arrow) (B, H&E stain, 100x). C. CMV immunostain highlights scattered positive cells (arrows) (200x). H&E: hematoxylin and eosin; CMV: cytomegalovirus

Histologic examination showed colonic mucosa with ulceration, cryptitis, crypt abscesses, dilated crypts with flattened epithelium, and focal crypt dropout, compatible with ICI-associated colitis. CMV immunohistochemical stain demonstrated scattered positive cells (Figure 2).

**Figure 3:**
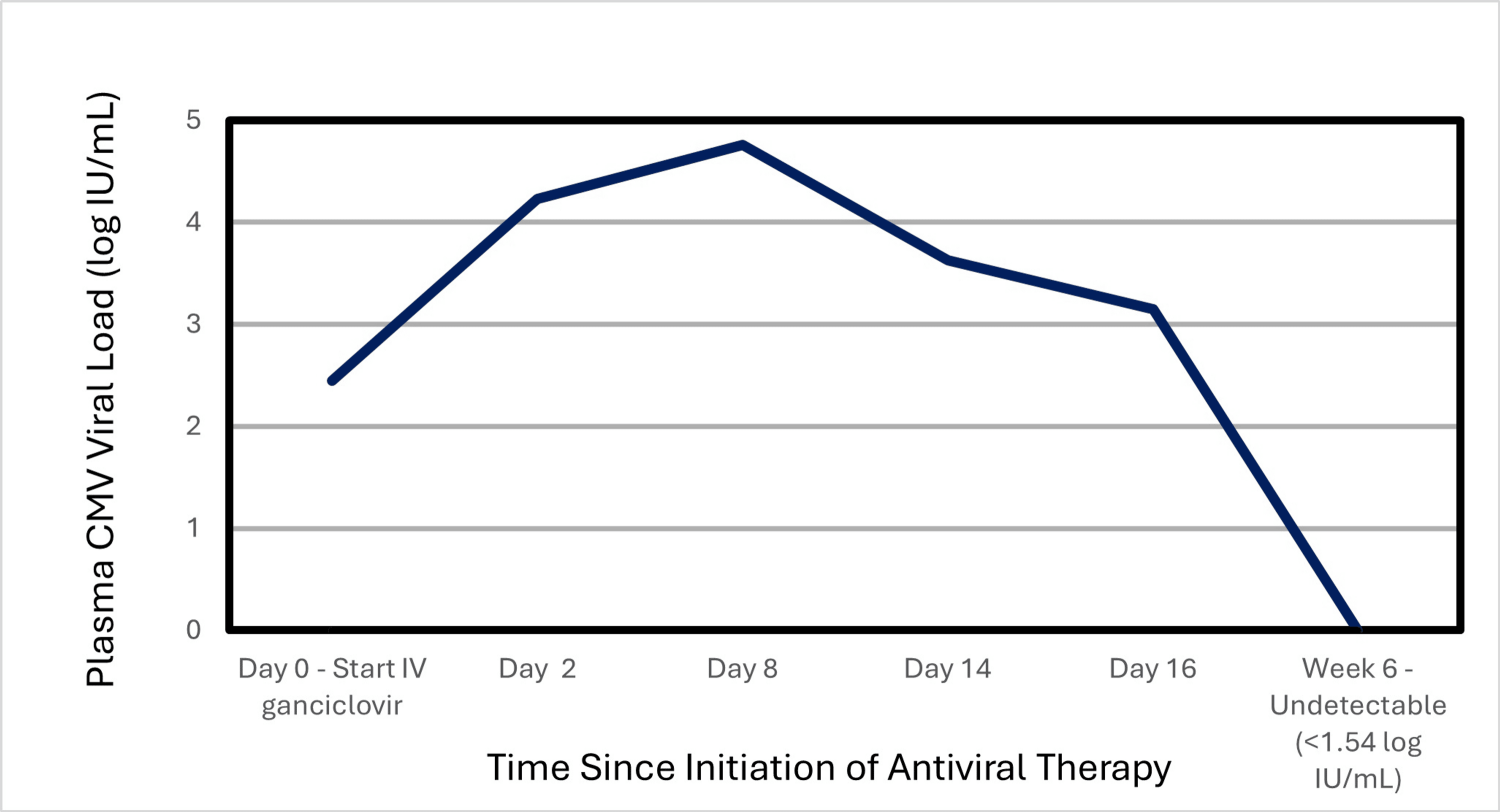
Serial plasma CMV DNA levels during antiviral therapy. Viral load initially increased following the diagnosis of CMV co-infection, peaked at 57,200 IU/mL (4.76 log IU/mL), and subsequently declined after intravenous ganciclovir therapy. CMV: cytomegalovirus

Plasma CMV DNA PCR obtained at the time of diagnosis was detectable at 283 IU/mL. Subsequently, the viral load increased to 57,200 IU/mL (4.76 log IU/mL) and declined after intravenous ganciclovir therapy. The patient’s diarrhea improved, and repeat testing six weeks later demonstrated undetectable CMV DNA, confirming sustained viral suppression (Figure 3).

## Discussion

ICI-associated colitis represents a heterogeneous pattern of mucosal injury caused by ICIs. A meta-analysis found that in patients with solid tumors treated with ICIs, the overall incidence of all-grade colitis was 9.1% for ipilimumab monotherapy, 13.6% for combination ipilimumab and nivolumab therapy, and 1.3% for PD-1/PD-L1 inhibitor monotherapy [3]. Combination ipilimumab and nivolumab therapy appears to have the highest incidence [3], and our patient was on this regimen as well. While many cases respond to therapy, a subset of patients can develop treatment-refractory disease [5]. In refractory cases, superimposed infection should be excluded before further immunosuppressive escalation as mucosal injury and ongoing immunosuppression increase susceptibility to opportunistic pathogens, which may complicate both diagnosis and management [5].

CMV is a well-recognized opportunistic pathogen in immunocompromised hosts. CMV colitis is defined by tissue-invasive viral disease, and identification of viral inclusions through routine histology or immunohistochemistry remains the gold standard for detection [6,7]. However, the pathogenic significance of CMV detection in inflamed mucosa remains debated. In inflammatory bowel disease, CMV may represent either a primary cause of inflammation or a secondary opportunistic infection in the setting of severe mucosal injury [7,8]. Similar uncertainty exists in ICI-associated colitis. Accurate diagnosis, therefore, requires integration of clinical, endoscopic, histologic, and virologic data. The endoscopic findings are often nonspecific, with diffuse inflammation that overlaps with immune-mediated and infectious colitis [9]. Histologic evaluation is critical to identify CMV-infected cells through identifying viral inclusions or through immunohistochemistry. Blood CMV-DNA polymerase chain reaction (PCR) can also provide supportive evidence of active viral replication [10].

In the present case, CMV-positive cells were detected by immunohistochemistry in a background of severe ICI-associated colitis. Serial blood quantitative PCR demonstrated an initial increase in CMV DNA levels, followed by virologic suppression after antiviral therapy. The declining CMV viral load correlated with the patient’s improvement in diarrhea, supporting that it was a biologically active infection rather than incidental viral detection. These findings suggest that CMV co-infection contributed to the clinical symptoms in this patient in the setting of refractory ICI-associated colitis.

## Conclusions

Our report showed the importance of maintaining CMV co-infection in the differential diagnosis for treatment-refractory ICI-associated colitis and highlighted the importance of clinicopathologic correlation in establishing the diagnosis. Integration of histologic findings with blood testing may assist in distinguishing incidental viral detection from clinically meaningful infection. However, viral suppression should not be equated with resolution of underlying ICI-associated colitis, and must be interpreted in the context of the overall clinical picture.
